# Risk Factors and Immunity in a Nationally Representative Population following the 2009 Influenza A(H1N1) Pandemic

**DOI:** 10.1371/journal.pone.0013211

**Published:** 2010-10-14

**Authors:** Don Bandaranayake, Q. Sue Huang, Ange Bissielo, Tim Wood, Graham Mackereth, Michael G. Baker, Richard Beasley, Stewart Reid, Sally Roberts, Virginia Hope, on behalf of the 2009 H1N1 serosurvey investigation team

**Affiliations:** 1 Institute of Environmental Science and Research, National Centre for Biosecurity and Infectious Disease, Upper Hutt, New Zealand; 2 Ministry of Health, Wellington, New Zealand; 3 Wellington School of Medicine, University of Otago, Wellington, New Zealand; 4 Medical Research Institute of New Zealand, Wellington Hospital, Wellington, New Zealand; 5 Ropata Medical Centre, Lower Hutt, New Zealand; 6 Department of Microbiology, Auckland District Health Board, Auckland, New Zealand; The University of Hong Kong, Hong Kong

## Abstract

**Background:**

Understanding immunity, incidence and risk factors of the 2009 influenza A(H1N1) pandemic (2009 H1N1) through a national seroprevalence study is necessary for informing public health interventions and disease modelling.

**Methods and Findings:**

We collected 1687 serum samples and individual risk factor data between November-2009 to March-2010, three months after the end of the 2009 H1N1 wave in New Zealand. Participants were randomly sampled from selected general practices countrywide and hospitals in the Auckland region. Baseline immunity was measured from 521 sera collected during 2004 to April-2009. Haemagglutination inhibition (HI) antibody titres of ≥1∶40 against 2009 H1N1 were considered seroprotective as well as seropositive. The overall community seroprevalence was 26.7% (CI:22.6–29.4). The seroprevalence varied across age and ethnicity. Children aged 5–19 years had the highest seroprevalence (46.7%;CI:38.3–55.0), a significant increase from the baseline (14%;CI:7.2–20.8). Older adults aged ≥60 had no significant difference in seroprevalence between the serosurvey (24.8%;CI:18.7–30.9) and baseline (22.6%;CI:15.3–30.0). Pacific peoples had the highest seroprevalence (49.5%;CI:35.1–64.0). There was no significant difference in seroprevalence between both primary (29.6%;CI:22.6–36.5) and secondary healthcare workers (25.3%;CI:20.8–29.8) and community participants. No significant regional variation was observed. Multivariate analysis indicated age as the most important risk factor followed by ethnicity. Previous seasonal influenza vaccination was associated with higher HI titres. Approximately 45.2% of seropositive individuals reported no symptoms.

**Conclusions:**

Based on age and ethnicity standardisation to the New Zealand Population, about 29.5% of New Zealanders had antibody titers at a level consistent with immunity to 2009 H1N1. Around 18.3% of New Zealanders were infected with the virus during the first wave including about one child in every three. Older people were protected due to pre-existing immunity. Age was the most important factor associated with infection followed by ethnicity. Healthcare workers did not appear to have an increased risk of infection compared with the general population.

## Introduction

The detection of the 2009 influenza A (H1N1) pandemic (2009 H1N1) virus in the United States and Mexico in April 2009, followed by widespread infection worldwide, prompted the World Health Organization (WHO) to declare the first pandemic in 41 years [1], [2], [3]. Non-seasonal influenza (capable of being transmitted between human beings) became a notifiable and quarantineable disease in New Zealand on 30 April 2009. From 1 April to 31 December 2009, a total of 3211 confirmed cases of 2009 H1N1 had been notified, including 1122 hospitalisations and 35 deaths [4]. Highest notification rates were seen in the under one year age group, and high notification and hospitalisation rates were seen among Pacific Peoples and Maori ethnic groups.

Estimating the true number of pandemic influenza cases in New Zealand from clinical surveillance is not possible as the vast majority of asymptomatic and mild symptomatic cases did not seek medical attention. Various models have been utilised to estimate the progress of the first wave of the pandemic but these have had to depend on imprecise assumptions as many key variables are unknown [5].

A serological measure of the population immunity profile in a community provides a truer picture of infection during the first wave, and allows for evidence-based decisions on interventions during future waves. A direct measure of neutralising antibodies to 2009 H1N1 before and after the first wave provides the cumulative incidence estimates of asymptomatic and symptomatic infections in a population, which could inform modelling initiatives for predicting subsequent pandemic waves [6]. Investigation of the potential risk factors of infection by analysis of information on host, environmental, behavioural and health service utilization factors obtained by a questionnaire would help guide public health interventions.

This report describes the first large nationally representative seroprevalence study from the southern hemisphere where 2009 H1N1 coincided with seasonal influenza infections. Immunity levels were measured in representative community participants and healthcare workers after the first wave of 2009 H1N1. The cumulative incidence of 2009 H1N1 was estimated by measuring neutralising antibodies to 2009 H1N1 using pre-pandemic (baseline) and post-pandemic serum samples. The risk factors for 2009 H1N1 were also analyzed by using information collected from questionnaires.

## Methods

### Ethics Statement

Ethics approval (MEC/09/09/106) was obtained from the Multiregional Ethics Committee of the New Zealand Ministry of Health. Written informed consent was obtained from all participants.

### Study design and population

Both community and healthcare worker studies involved a multi-stage random cross-sectional design and a questionnaire evaluating demographics and potential risk factors.

#### Community study

The study population consisted of the registered patients enrolled in the selected general practitioner (GP) clinics and were individuals residing in New Zealand before, during and after the first wave of the pandemic. Random samples of patients stratified by age and ethnicity were obtained from the study population during the period November 2009 to March 2010. Serological results from these samples reflected immunity acquired during the first wave from April to September 2009 as well as any pre-existing immunity.

The first stage of the cross-sectional study was a purposive cluster sample of general practices, followed by a stratified random sample of registered patients. The study included 14 GP clinics across the country. The study localities were selected in predetermined areas based on observed incidence during the pandemic as high, medium and low, as well as the ethnic distribution. Practices already participating in the on-going national sentinel surveillance system for influenza were preferred. Within each practice, registered patients were stratified by age group and ethnicity. Five age groups were categorised as 1 to 4, 5 to 19, 20 to 39, 40 to 59, and ≥60. Ethnicity was recorded according to New Zealand Census categories, but for analytical purposes was divided into three categories as Maori, Pacific Peoples and Other.

Within each stratum, simple random sampling was performed to select sufficient numbers of participants. Taking into account stratification, a minimum sample size of 1500 participants was required, at design prevalence of 20% and confidence level (CI) of 95%, to maintain +/−10% acceptable margin error of the estimate.

Following random selection from GP registers with purposive sampling of GP practices to meet strata requirements, telephone contact with a participant was made and a questionnaire was administered to collect exposure and risk factor information. The questionnaire included information on the participant's demographics, history of influenza-like illness (ILI) and other acute illnesses, contact with ILI patients, general health status, vaccination history, and living conditions. Information sheets, consent forms and blood sample request forms were made available to the participants. The expected low response from minority ethnic groups and very young children, was counteracted by systematic recruitment during consultations in three GP clinics and the use of finger-prick samples respectively. A 5 ml venous or finger-prick blood sample was collected and transported to the WHO National Influenza Centre (NIC) at Institute of Environmental Science and Research (ESR) for haemmagglutination inhibition testing. In total, 1156 participants were enroled in the study. Nine participants did not return the questionnaires and thus were excluded from this analysis. This gave an overall response rate of 76% (1147/1500).

#### Healthcare worker study

The study population included secondary healthcare workers (HCWs) located in Auckland and Middlemore hospitals and primary HCWs from the 14 GP practices included in the community study. HCWs were divided into three categories as medical, nursing, and other staff (including allied health and support staff). A simple random sampling procedure was performed to select sufficient numbers of participants. In total 171 primary HCWs and 369 secondary HCWs were enroled during January to March 2010.

#### Baseline study

The baseline immunity to 2009 H1N1 was measured from 521 serum samples taken before 22-April 2009 from individuals aged 1 to 98 years. 184 sera, collected from children aged 1–19 years during 2004–2005, were obtained from ESR's Invasive Pathogen Laboratory for the purpose of the meningococcal seroprevalence study. 337 sera, collected from adults aged ≥20 years during 2004 to 22-April 2009, mainly for the serological testing for arboviruses and polioviruses, were obtained from ESR's Clinical Virology Reference Laboratory. These were residual samples submitted to the laboratories for diagnostic testing or antibody screening. Only information about age, sex, sample collection date and collecting laboratory was available for these samples.

### Laboratory Method

Antibodies against 2009 H1N1 were detected by using haemmagglutination inhibition (HI) assay, according to standard methods [7], [8]. The HI assay used 1.0% guinea pig erythrocytes. A reference antigen, pandemic influenza A/California/7/2009 virus propagated in embryonated chicken eggs, was provided by WHOCC-Melbourne. Serial two-fold dilutions of serum were tested beginning with a 1∶10 dilution and a final dilution of 1∶1280. Suitable control serum samples were included in all assays, including post-infection ferret serum samples raised against the A/Auckland/1/2009 strain and a known human sample with a known HI titre as a positive control. All human sera samples were treated with receptor destroying enzyme (Vibrio Cholera Neuraminidase) and guinea-pig erythrocytes to inactivate non-specific inhibitors of viral haemagglutination. The antibody level was measured as the titre of heamagglutination inhibition. The reciprocal of the highest dilution causing complete haemagglutination inhibition of erythrocytes by the 2009 H1N1 virus was used as a measure of the antibody level to the pandemic virus. It has been shown that susceptibility to influenza virus infection is inversely related to the initial titre of serum HI antibody. HI antibody titres of ≥1∶40 are correlated with a reduction of 50% of the risk of contracting an influenza infection or disease [9], [10], [11], [12]. Thus, in this study, an HI titre of 1∶40 is used as the threshold of seroprotection as well as seropositivity. The proportion of individuals with HI titres of ≥1∶40 against 2009 H1N1 in the serosurvey is referred to as seroprevalence. Geometric mean titres (GMTs) were estimated by assigning a value of 1∶10 for titres of 1∶10 or lower and a value of 1∶1280 for titres of 1∶1280 or higher.

### Data analysis and statistics

For the community seroprevalence study, individuals have had different probabilities of being selected for the sample, due to stratification and unequal allocation. Therefore, stratified and weighted analysis was performed to account for the study design. Rao-Scott Chi-squares test, which is a design-adjusted version of the Pearson chi-square test, was used to test the significance of the estimates at p value equal to 0.05.

#### Descriptive analysis

We performed descriptive analysis for the categorical and numerical data using PROC SURVEYFREQ and PROC SURVEYMEANS of SAS 9.1 version (SAS Institute Inc., Box 8000, Cary, NC), respectively. These procedures allow incorporating the sample design by specifying the age-ethnicity strata and sampling weights.

#### Multivariable analysis

The main hypothesis being tested in this analysis was whether age group or ethnicity affected the likelihood of 2009 H1N1 immunity. The final model included age group, ethnic group, sex, vaccination history, chronic illness, reported damp housing, and study area as explanatory variables, with 2009 H1N1 seroprotective result as the outcome. Multivariable survey logistic regression was the method of choice since the outcome was binary (1 =  evidence of infection to 2009 H1N1, 0 =  no evidence). This analysis included 820 of the 1147 participants, who had complete information for age, ethnicity, serology results, and for selected risk factors.

Univariable screening analysis for inclusion was done at P≤0.2. Variables associated with seropositive test at P≤0.2 were then included into a multivariable survey logistic regression model. Pearson correlation was performed to assess the correlation between risk factors. If factors were significantly correlated, then only one of these was selected for the model. Variables were allowed to remain in the model if statistically significant at P<0.05 using stepwise selection, with seropositive status (0/1) as the dependent variable. Potential confounders such as housing condition and seasonal vaccination history were forced into the model. Interaction terms were constructed from main effect variables and tested for significance. The final model included age group, ethnic group and sex, vaccination history, chronic illness, and reported damp housing as independent variables. Since damp housing correlated with those also reporting cold or musty housing conditions, we used the former in our model. Statistical analyses were performed using SAS version 9.1 (SAS Institute Inc., Box 8000, Cary, NC).

## Results

### Characteristics of the sample

Table 1 shows demographis characteristics of the samples collected for the community, healthcare workers and baseline study.

**Table 1:** Sample demographics for the community, healthcare worker, and baseline study.

	Community Study		Healthcare workers		Baseline	
Demography	Number of samples	Percent (%)	Number of samples	Percent (%)	Number of samples	Percent (%)
**Age group (years)**						
1 to 4	152	13.2			84	16.1
5 to 19	209	18.1			100	19.2
20 to 39	221	19.2	238	44.2	106	20.4
40 to 59	258	22.4	250	46.4	107	20.5
60 and over	314	27.2	51	9.5	124	23.8
**Ethnic group**						
Maori	184	15.9	24	4.6		
Pacific	171	14.8	18	3.4	Not Available	
Other	801*	69.3	485	92.0		
**Gender**						
Female	640	55.6	436	80.7	176	52.4
Male	511	44.4	104	19.3	160	47.6
**Study area**						
Auckland	269	23.3	423	78.3		
Waikato	107	9.3				
Bay of Plenty	122	10.6	18	3.3		
MidCentral	113	9.8			Not Available	
Wellington	370	32.0	78	14.4		
Canterbury	109	9.4				
Otago	66	5.7	21	3.9		
**Overall**	**1156**		**540**		**521**	

*Including 457 Europeans only.

### Seroprevalence in study populations

We analyzed serology results for the 1147 community participants, 532 healthcare workers, and 521 baseline samples (Table 2). The overall community seroprevalence was 26.7% (CI:23.4–29.9). Seroprevalence varied across age groups. School aged children (5–19 years) had the highest seroprevalence (46.6%;CI:38.3–54.9), a significant increase from the baseline (14%;CI:7.2–20.8). Older adults aged ≥60 had no significant difference in seroprevalence between the serosurvey (24.8%;CI:18.7–30.9) and baseline (22.6%;CI:15.3–30.0). Pacific Peoples had significantly higher seroprevalence (49.5%;CI:34.9–64.2) than Maori and Other (Europeans etc) ethnic groups. There were no statistically significant difference in seroprevalence by sex or by study areas. The seroprevalence of primary HCWs (29.3%;CI:22.4–36.3) and secondary HCWs (25.3%;CI:20.8–29.8) showed no significant difference from the community participants aged 20–59 years (21%;CI:16.5–25.7). There was no difference in seroprevalence among doctors (29.9%;CI:21.9–37.9), nurses (27.5%;CI:21.3–33.7) and support staff (25.0%;CI:18.7–31.3). Based on age and ethnicity standardization to the national population, an estimated 29.5% of New Zealanders (1.3 million) had immunity to 2009 H1N1. Based on the questionnaire survey approximately 45.2% (CI:38.0–52.4) of seropositive individuals had had no symptoms. This percentage did not change to any appreciable extent following different calculations where the seropositive individuals with pre-existing immunity were excluded and those with symptoms due to pathogens other than 2009 H1N1 were included.

**Table 2:** 2009 H1N1 seroprevalence in the community, healthcare workers, and baseline samples.

Sero-survey	No. Tested	No. Sero Positive (Titre >40)	Seroprevalence	P-value for group
			(95% CI)	
Overall*	1147	347	26.7 (23.4–29.9)	
**Age group (years)** 1				<0.001
1 to 4	148	55	29.5 (21.0–38.0)	
5 to 19	206	102	46.7 (38.3–55.0)	
20 to 39	221	61	22.2 (15.6–28.9)	
40 to 59	258	56	20.2 (14.0–26.5)	
60 and over	314	73	24.8 (18.7–30.9)	
**Ethnic group** 2				0.001
Maori	181	62	36.3 (28.0–44.6)	
Pacific	167	73	49.5 (35.1–64.0)	
Other	799	212	25.9 (22.4–29.4)	
**Sex** *				0.94
Female	636	194	26.5 (22.2–30.9)	
Male	506	152	26.8 (21.8–31.8)	
**Study area** *				0.36
Auckland	262	82	23.6 (16.3–30.8)	
Waikato	107	22	20.0 (10.2–29.7)	
Bay of Plenty	122	38	27.7 (18.5–36.9)	
MidCentral	113	36	26.4 (16.8–36.0)	
Wellington	369	117	30.2 (24.5–36.0)	
Christchurch	109	32	19.4 (11.1–27.7)	
Otago	65	20	29.4 (16.8–41.9)	
**Healthcare workers**				
Primary	169	50	29.6 (22.6–36.5)	
Secondary	363	92	25.3 (20.8–29.8)	
**Occupation**				>0.05
Doctor	127	38	29.9 (21.9–37.9)	
Nurse	200	55	27.5 (21.3–33.7)	
Other	184	46	25.0 (18.7–31.3)	
**Baseline immunity**				
Overall	521	62	11.9 (9.1–14.7)	
**Age group (Years)**				<0.001
1 to 4	84	5	6.0 (0.9–11.0)	
5 to 19	100	14	14.0 (7.2–20.8)	
20 to 39	106	8	7.5 (2.5–12.6)	
40 to 59	107	7	6.5 (2.0–11.1)	
60 and over	124	28	22.6 (15.3–30.0)	

P-value calculated using the Rao-Scott chi-square test.

1 Ethnicity-adjusted estimates for the study population.

2 Age-adjusted estimates for the study population.

*Age- and ethnicity-adjusted overall estimate.

### Determinants of immunity

Responses to the questionnaires from community participants were analyzed to identify deteminants of immunity. These included a range of host, environmental, behavioural and health service utilization factors (Table S1). Most factors showed no association with increased immunity. Large household size (>4 people) was associated with higher immunity (Crude OR,1.6;CI:1.11–2.31, p = 0.011) compared to small household size (1–4 people). However, after the age adjustment, this effect was no longer present (Adjusted OR,1.36;CI:0.92–2.03, p = 0.126).

**Figure 1:**
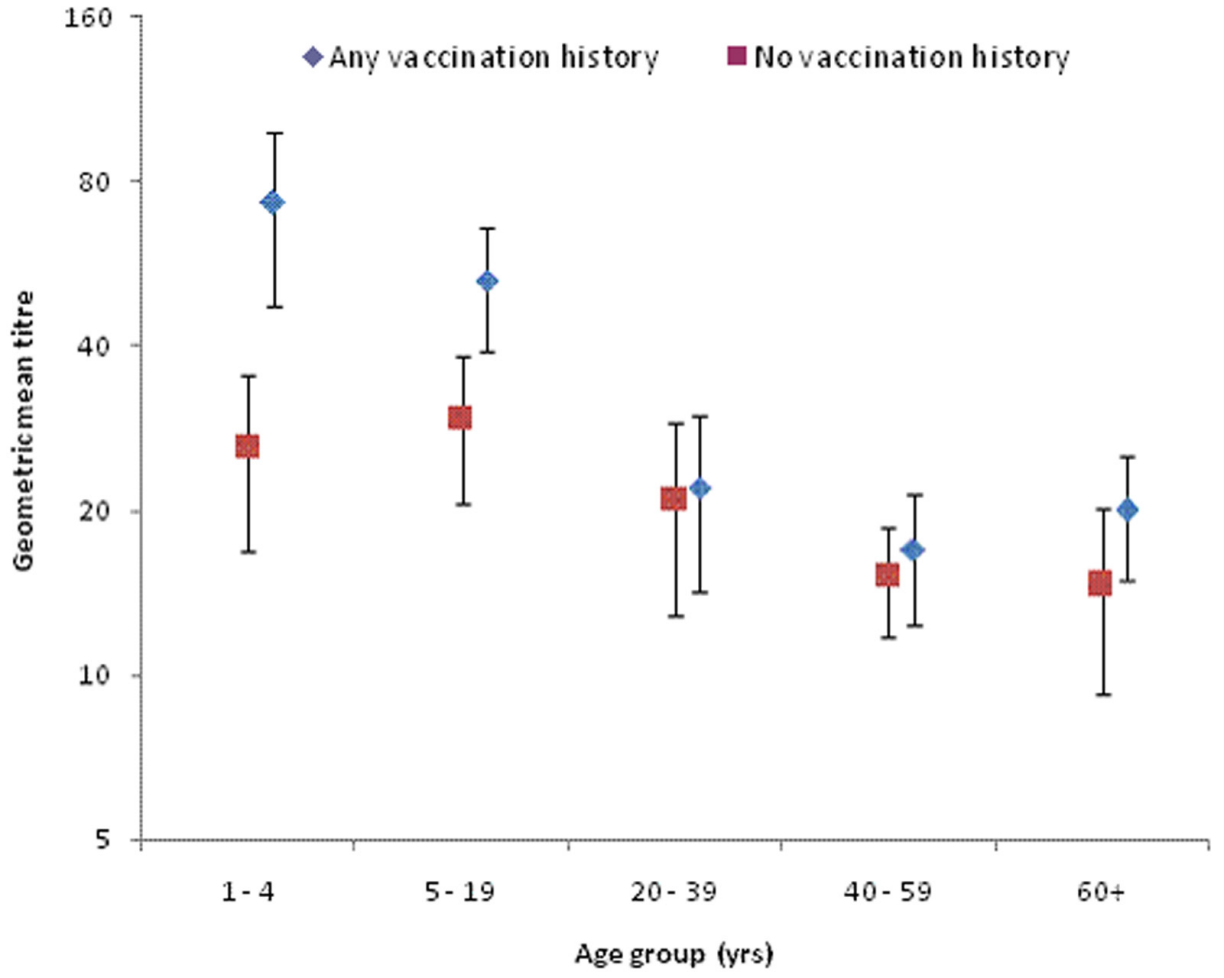
Effect of seasonal influenza vaccination on geometric mean titres by age groups in the community study.

Individuals with previous seasonal influenza vaccination showed higher geometric mean titres than those without vaccinations, particularly in children aged 1–4 and 5–19 years (Figure 1).

Multivariate analysis was performed to test whether age group and ethnicity affected 2009 H1N1 immunity level. Table 3 shows the ouputs from the multivariable survey logistic model. Younger age groups were associated with an increased likelihood of immunity. The likelihood of 2009 H1N1 immunity among the age group 5–19 and age group 1–4, respectively, was 5.3 (CI:3.2–8.7 p<0.001) times and 3.5 (CI:2.0–6.2 p = 0.029) times higher compared with that of age group 40–59 (the reference group). The likelihood of 2009 H1N1 immunity was 2.2 (CI:1.5–3.4 p<0.001) times higher in the Pacific People compared with that of the “Other” ethnic group (the reference group). These results have confirmed the findings in the descriptive analysis as shown by the ethnicity effect (p = 0.009). Participants with previous seasonal influenza vaccinations were 1.8 times (p = 0.002) more likely to have HI titers of ≥1∶40 compared with those who had never been vaccinated.

**Table 3:** Results from the multivariate survey logistic regression model for selected factors.

Risk factors	Odds Ratio for antibody titer ≥1∶40	Lower CI	Higher CI	P - value
*Age group (years)*				
1 to 4	3.5	2	6.2	<0.001
5 to 19	5.3	3.2	8.7	<0.001
20 to 39	1.4	0.85	2.3	0.18
40 to 59	Reference	-	-	-
60 and over	0.95	0.6	1.5	0.84
*Ethnic group*				
Maori	1.4	0.95	2.2	0.09
Pacific	2.2	1.5	3.4	<0.001
Other	Reference	-	-	-
Sex (male/female)	0.82	0.59	1.1	0.21
Any vaccination history (yes/no)	1.8	1.2	2.6	0.002
Prior chronic illness (yes/no)	1.2	0.81	1.7	0.41
Damp housing (yes/no)	1.1	0.83	1.4	0.62

**Figure 2:**
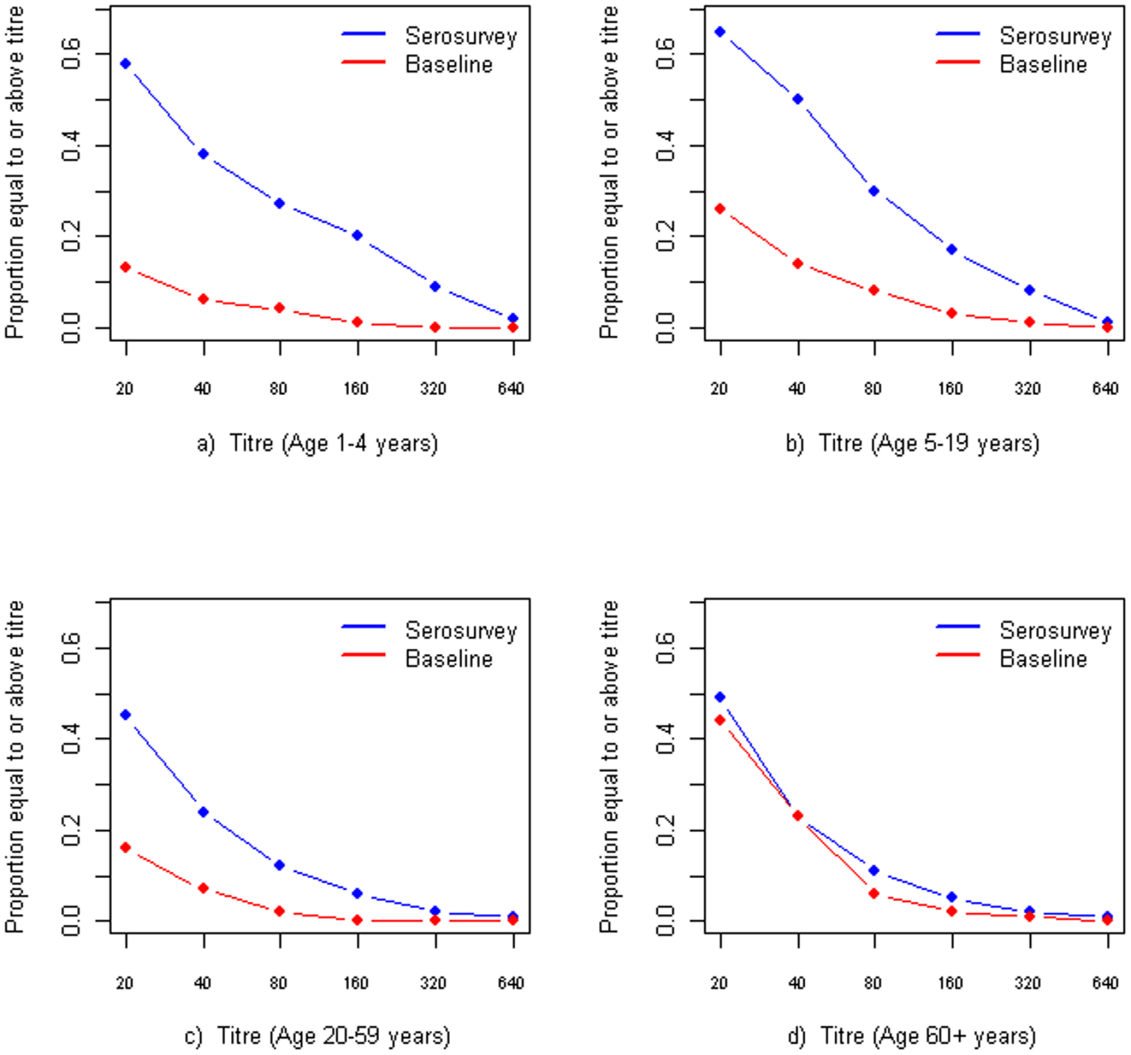
Proportions of the baseline and serosurvey samples equal to or above each titre level for the age groups of 1–4 (a), 5–19 (b), 20–59 (c) and ≥60 years (d).

The difference in the proportion of seroprotective individuals from the baseline and the serosurvey of 2009 H1N1 was considered as a proxy measure of the cumulative incidence of infection due to the pandemic virus [13]. Figure 2 showed proportions of the baseline and serosurvey samples equal to or above each titre level for age groups of 1–4, 5–19, 20–59 and ≥60 years. Children in 1–4 and 5–19 years showed significant differences between the baseline and serosurvey at almost every titre level while older adults had little difference at any titre level.

### Infection and health impact of the 2009 H1N1 pandemic

The difference in the proportion of individuals with HI titre of ≥1∶40 was compared between the baseline and serosurvey samples among different age groups. Based on age and ethnicity standardization to the national population, an estimated 18.3% of New Zealanders were infected with 2009 H1N1. Our population cumulative incidence estimates (781,867 cases) are substantially higher than the case estimates from various clinical surveillance data [4], [14]. Based on the questionnaire survey, a substantial proportion of seropositive individuals (45.2%;CI:38.0–52.4) did not report any symptoms while 54.8% (CI:47.6–62.0) of seropositive individuals reported at least one symptom. This gives an estimated total of 428,463 symptomatic cases. Taking symptomatic case and infected case estimates from the serosurvey and the known number of deaths (35), the case fatality ratio was 8.2 per 100,000 (0.008%, 35/428,463) of symptomatic cases and 4.5 per 100,000 (0.004%, 35/781,867) of infected cases. Using hospital admission as an indicator of severity and the known number of admissions (1122), the hospitalization ratio was 262 per 100,000 (0.262%, 1122/428,463) of symptomatic cases and 144 per 100,000 (0.144%, 1122/781,867) of infected cases.

## Discussion

To our knowledge, this is the first nationally representative serological study from a temperate southern hemisphere country. It provides useful information on the population immunity profile in New Zealand and new insights into the epidemiology of the pandemic virus infection during the first wave. This study used a simple and replicable design which produced adequate response rates while minimizing the in-built bias inherent in other seroprevalence studies utilizing non-random samples. Furthermore, this survey allows analysis of information on potential contributing factors to 2009 H1N1 infection, which will inform future public health interventions.

The highest proportion of individuals with protective immunity and pandemic virus infection was found in school aged children (5–19 years) at 46.7% with a significant increase of 32.7% from the baseline immunity of 14.0%. Our study showed a higher infection rate with the pandemic virus in the school age children. This finding accords with the notion that school age children constitute the main conduit for spread of influenza, probably due to generally higher levels of contact in school. In this respect our results were very similar to the findings reported from several other developed countries [13], [15], [16].

A high proportion (22.6%) of older adults aged ≥60 years had cross-reactive antibodies against 2009 H1N1 before the first wave. Older adults could acquire immunity to 2009 H1N1 virus, as a result of previous exposure to a 1918-like A(H1N1) virus circulating during 1918–1957, or a lifetime of exposure to influenza A, which has resulted in broad heterotypic immunity [12], [17], [18], [19], [20]. This pre-existing immunity is consistent with clinical surveillance reported in New Zealand where pandemic cases were concentrated in younger age groups [14], [21]. Older adults (24.8%) had HI titers of ≥1∶40 in the serosurvey with little increase from the baseline and no increase in GMT. However, we only assessed neutralizing antibody against 2009 H1N1 haemagglutinin in this study. It is possible that heterotypic immunity to influenza from antibody against the neuraminidase or cellular responses to highly conserved viral epitopes might have also contributed to the apparent protective effect in older adults [22]. Further study on the effect of heterotypic immunity on age-specific populations is needed.

An overall low proportion of children and adults (1–59 years) had cross-reactive antibodies to 2009 H1N1 in the baseline samples, similar to the British and Victoria reports [13], [23]. However, 6% of children aged 0–4 years had HI titers of ≥1∶40 in the baseline samples, higher than 1.8% reported by the British study [13], but not significantly different. This difference may reflect the varied influenza exposure these young children experienced in the two countries. Also, the relatively small numbers of opportunistic diagnostic sera in the baseline samples were collected without randomization and with no information on seasonal influenza vaccination and other important determinants. This is one of the limitations of this study regarding the representativeness of baseline samples across all age groups. Random sampling of the population for the baseline would have been ideal.

Pacific Peoples had the highest seroprevalence followed by those of Maori origin. Pacific and Maori peoples also had much higher hospitalization and intensive care unit admission rates compared with European and other groups [4], [14]. The younger population age structure in Pacific and Maori peoples does not fully explain these ethnic groups' apparent susceptibility to 2009 H1N1. Other contributing factors to these ethnic differences may include: higher prevalence of the infection in Pacific and Maori peoples: higher prevalence of co-morbidities (such as asthma and diabetes), unfavorable environmental factors (such as household crowding and poor quality housing), behavioral differences in responses to influenza, differences in socio-cultural-economic status, differences in health service utilization and increased genetic susceptibility [24]. Further study on the contributing factors to ethnic differences in the risk of 2009 H1N1 infection and severe disease is underway in New Zealand.

The seroprevalence among primary and secondary healthcare workers did not differ significantly compared with that of the general population. In the Taiwanese study, the front-line hospital workers (20% seroprevalence with mean ages of 36.9±10.6) is significantly higher than the the general population (less than 3% with mean ages of 52.0±12.6 years), which may reflect a higher contact risk [25]. In this context it is likely that the standard infection control measures routinely used in New Zealand HCWs provide adequate protection even though such measures do not include pre-exposure antiviral prophylaxis. In addition, there was no significant difference in seroprevalence between doctors, nurses and support staff. Further study with individualised information regarding risk exposures and personal protective measures is needed.

The difference in the proportion of individuals with HI titre of ≥1∶40 between the baseline and serosurvey among different age groups, was considered an appropriate proxy measure of the cumulative incidence of infection due to 2009 H1N1 [13]. There are some limitations associated with this measure. Firstly, cumulative incidence estimates required comparison of the proportion of neutralizing antibodies against 2009 H1N1 before and after the pandemic, which reduces the precision of the estimate for a given sample. Secondly, this measure may lead to an underestimate of infection by 2009 H1N1 because the threshold (HI titre of 1∶40) may underestimate the true proportion of individuals who were infected. Thirdly, this measure assumes that all age groups respond to the pandemic virus in the same way immunologically. This is a simplified assumption for a complex host immunological response that may vary across age groups. Our findings suggested that 2009 H1N1 triggered different response in titers of neutralizing antibodies in different age groups. Lastly, this measure may underestimate cumulative incidence for individuals who were infected with 2009 H1N1 but never developed HI antibodies. Further studies are needed to define a serological marker of infection specific to 2009 H1N1 that do not detect cross-reactive antibodies to other seasonal influenza A(H1N1) viruses.

The high proportion of seropositive individuals that did not report illness gives an indication of a relatively ‘silent’ spread of the disease in any naive population. While asymptomatic individuals may be less infective, their role in the spread of 2009 H1N1 cannot be discounted. This finding has important implications for public health policy measures that were instituted at ports of entry and educational institutions during the first wave of the pandemic. It underscores the need for vigilance both at the community and individual levels to reduce the spread of disease. Basic hygiene measures such as regular hand-washing become important whether or not one has a conspicuous ILI.

Our serosurvey showed that previous seasonal influenza vaccination was associated with higher HI titers against 2009 H1N1, similar to the findings in other reports [22] [26]. Hancock et al analyzed stored-serum samples from trials of seasonal trivalent inactivated vaccines predating the 2009 pandemic and showed the presence of cross-reactive antibodies to 2009 H1N1 in adults and very little response in children [22]. The same study showed that vaccination with the seasonal vaccine resulted in a doubling in titers of these cross-reactive antibodies to 2009 H1N1. Interestingly, our study also showed that participants with any previous seasonal influenza vaccination were about twice (1.8 times) more likely to have HI titers of ≥1∶40 against 2009 H1N1 compared with those who have never been vaccinated. Two possible mechanisms can be used to explain this observation. Firstly, it was assumed the participants with or without seasonal influenza vaccines had a similar probability to be infected with the pandemic virus. Compared to the non-vaccinated participants, the vaccinated participants would respond with higher HI titres when infected with the pandemic virus because they had been primed with seasonal influenza vaccines previously. Secondly, recipients of seasonal influenza vaccines may have had an increased risk of contracting 2009 H1N1 compared to non-recipients. This confounding would result in higher HI titres in the vaccinated participants than in the non-vaccinated ones. Several studies have examined the effectiveness of seasonal influenza vaccine against 2009 H1N1. The case-control study from Australia [27], the case-cohort study from USA [28] and the outbreak investigation in New York City [29] did not support a significant effect of 2008-09 trivalent influenza vaccine in either decreasing or increasing the risk for 2009 H1N1 illness. In addition, investigators from Mexico conducted a hospital-based case-control study and reported a vaccine effectiveness of 73% (CI = 34% to 89%) from the 2008-09 trivalent inactivated vaccine against 2009 H1N1 illness [30]. Conversely, a series of five studies conducted in four Canadian provinces reportedly found that receipt of seasonal 2008-09 influenza vaccine was associated with a 1.5- to 2-fold greater risk for medically attended 2009 H1N1 illness [31]. Further research is needed to evaluate the effects of seasonal influenza vaccination on infection with 2009 H1N1.

Vaccination strategies include targeting people at risk of adverse health outcomes and boosting population immunity to prevent transmission. Our findings can help public health authorities to make evidence-based decisions on vaccination strategies and priority listing for 2009 H1N1 and future pandemics. For example, while children 5–19 years who played an important role in the community transmission of infection are now largely protected against 2009 H1N1, they could be a high priority for pandemic influenza vaccination in the event of another novel pandemic strain.

## Supporting Information

Table S1 Univariate and age-adjusted analysis for selected health determinants of immunity in community participants.
(0.03 MB DOC)

## References

[1] CDC (2009). Update: swine influenza A (H1N1) infections–California and Texas, April 2009.. MMWR Morb Mortal Wkly Rep.

[2] WHO Swine Influenza - Update 3. Available: http://www.who.int/csr/don/2009_04_27/en/index.html Accessed on 22 January 2010

[3] Chan M (2009). http://www.who.int/mediacentre/news/statements/2009/h1n1_20090425/en/index.html.

[4] Lopez L, Huang QS (2009). http://www.surv.esr.cri.nz/virology/influenza_annual_report.php.

[5] Paine S, Mercer G, Kelly P, Bandaranayake D, Baker M (2010). Transmissibility of 2009 pandemic influenza A(H1N1) in New Zealand: effective reproduction number and influence of age, ethnicity and importations.. Euro Surveill.

[6] Lipsitch M, Hayden FG, Cowling BJ, Leung GM (2009). How to maintain surveillance for novel influenza A H1N1 when there are too many cases to count.. The Lancet.

[7] Kendal A, Pereira M, Skehel J (1982). Concepts and procedures for laboratory-based influenza surveillance..

[8] Rowe T, Abernathy RA, Hu-Primmer J, Thompson WW, Lu X (1999). Detection of antibody to avian influenza A (H5N1) virus in human serum by using a combination of serologic assays.. J Clin Microbiol.

[9] Hobson D, Curry RL, Beare AS, Ward-Gardner A (1972). The role of serum haemagglutination-inhibiting antibody in protection against challenge infection with influenza A2 and B viruses.. J Hyg (Lond).

[10] Potter CW, Oxford JS (1979). Determinants of immunity to influenza infection in man.. Br Med Bull.

[11] de Jong JC, Palache AM, Beyer WE, Rimmelzwaan GF, Boon AC (2003). Haemagglutination-inhibiting antibody to influenza virus.. Dev Biol (Basel).

[12] Potter CW, Jennings R, Nicholson K, Tyrrell DA, Dickinson KG (1977). Immunity to attenuated influenza virus WRL 105 infection induced by heterologous, inactivated influenza A virus vaccines.. J Hyg (Lond).

[13] Miller E, Hoschler K, Hardelid P, Stanford E, Andrews N (2010). Incidence of 2009 pandemic influenza A H1N1 infection in England: a cross-sectional serological study.. Lancet.

[14] Baker MG, Wilson N, Huang QS, Paine S, Lopez L (2009). Pandemic influenza A(H1N1)v in New Zealand: the experience from April to August 2009.. Euro Surveill.

[15] Zimmer SM, Crevar CJ, Carter DM, Stark JH, Giles BM (2010). Seroprevalence following the second wave of Pandemic 2009 H1N1 influenza in Pittsburgh, PA, USA.. PLoS One.

[16] Ross T, Zimmer S, Burke D, Crevar C, Carter D (2010). Seroprevalence Following the Second Wave of Pandemic 2009 H1N1 Influenza.. PLoS Curr Influenza.

[17] Greenberg SB, Couch RB, Kasel JA (1974). An outbreak of an influenza type A variant in a closed population: The effect of homologous and heterologous antibody on infection and illness.. Am J Epidemiol.

[18] Larson HE, Tyrrell DA, Bowker CH, Potter CW, Schild GC (1978). Immunity to challenge in volunteers vaccinated with an inactivated current or earlier strain of influenza A(H3N2).. J Hyg (Lond).

[19] Pereira MS, Chakraverty P, Schild GC, Coleman MT, Dowdle WR (1972). Prevalence of antibody to current influenza viruses and effect of vaccination on antibody response.. Br Med J.

[20] Potter CW, Jennings R, Phair JP, Clarke A, Stuart-Harris CH (1977). Dose-response relationship after immunization of volunteers with a new, surface-antigen-adsorbed influenza virus vaccine.. J Infect Dis.

[21] Huang QS, Bandaranayake D, Lopez LD, Pirie R, Peacey M (2009). Surveillance for the 2009 pandemic influenza A (H1N1) virus and seasonal influenza viruses - New Zealand, 2009.. MMWR Morb Mortal Wkly Rep.

[22] Hancock K, Veguilla V, Lu X, Zhong W, Butler EN (2009). Cross-reactive antibody responses to the 2009 pandemic H1N1 influenza virus.. N Engl J Med.

[23] Grills N, Piers LS, Barr I, Vaughan LM, Lester R (2010). A lower than expected adult Victorian community attack rate for pandemic (H1N1) 2009.. Aust N Z J Public Health.

[24] La Ruche G, Tarantola A, Barboza P, Vaillant L, Gueguen J (2009). The 2009 pandemic H1N1 influenza and indigenous populations of the Americas and the Pacific.. Euro Surveill.

[25] Chan YJ, Lee CL, Hwang SJ, Fung CP, Wang FD (2009). Seroprevalence of Antibodies to Pandemic (H1N1) 2009 Influenza Virus Among Hospital Staff in a Medical Center in Taiwan.. Journal of the Chinese Medical Association.

[26] Chen MI, Lee VJ, Lim WY, Barr IG, Lin RT (2010). 2009 influenza A(H1N1) seroconversion rates and risk factors among distinct adult cohorts in Singapore.. JAMA.

[27] Kelly H, Grant K (2009). Interim analysis of pandemic influenza (H1N1) 2009 in Australia: surveillance trends, age of infection and effectiveness of seasonal vaccination.. Euro Surveill.

[28] Gargiullo P, Shay D, Katz J, Bramley A, Nowell M (2009). Effectiveness of 2008-09 trivalent influenza vaccine against 2009 pandemic influenza A (H1N1) - United States, May-June 2009.. MMWR Morb Mortal Wkly Rep.

[29] Iuliano AD, Reed C, Guh A, Desai M, Dee DL (2009). Notes from the field: outbreak of 2009 pandemic influenza A (H1N1) virus at a large public university in Delaware, April-May 2009.. Clin Infect Dis.

[30] Garcia-Garcia L, Valdespino-Gomez JL, Lazcano-Ponce E, Jimenez-Corona A, Higuera-Iglesias A (2009). Partial protection of seasonal trivalent inactivated vaccine against novel pandemic influenza A/H1N1 2009: case-control study in Mexico City.. Br Med J.

[31] Skowronski DM, De Serres G, Crowcroft NS, Janjua NZ, Boulianne N (2010). Association between the 2008-09 seasonal influenza vaccine and pandemic H1N1 illness during Spring-Summer 2009: four observational studies from Canada.. PLoS Med.

