# Supplementary material for: Risk Factors and Immunity in a Nationally Representative Population following the 2009 Influenza A(H1N1) Pandemic
Source: PLoS One. 2010 Oct 14;5(10):e13211. doi: 10.1371/journal.pone.0013211 (PMC2954793; doi:10.1371/journal.pone.0013211)
Supplement: Table S1 — Univariate and age-adjusted analysis for selected health determinants of immunity in community participants. (0.03 MB DOC) [file pone.0013211.s001.doc]

Table S1 Univariate and age-adjusted analysis for selected health determinants of immunity in community participants

*A proxy measure of socioeconomic status derived from the participant’s home address and measured on an ordinal scale of 1 to 10 where 1 indicates the individual is from the least deprived 10th decile area in New Zealand [Salmond C, Crampton P (2002) NZDep2001 index of deprivation. Available: http://www.moh.govt.nz/moh.nsf/Files/NZDepfiles/$file/phi-users-manual.pdf. Accessed June 31, 2010]

^1^ Binary variable (yes, no) with “no” as the reference category
